# Efficacy and Response of R21/Matrix-M in Malaria Vaccine: A Systematic Review and Meta-Analysis of Clinical Trials

**DOI:** 10.3390/vaccines14090824

**Published:** 2026-09-19

**Authors:** Aneeq Ur Rehman, Victory Nnaemeka, Muhammad Hassan Nasir, Nurul Alia Azizan, Ahmed M. Salman, Ahmad Syibli Othman

**Affiliations:** 1 School of Biomedical Sciences, Faculty of Health Sciences, Universiti Sultan Zainal Abidin, Kuala Nerus 21300, Malaysia; 2 The Jenner Institute, Nuffield Department of Medicine, University of Oxford, Oxford OX3 7DQ, UK; 3 Faculty of Medicine, Universiti Sultan Zainal Abidin, Kuala Terengganu 20400, Malaysia

**Keywords:** R21/Matrix-M vaccine, vaccine efficacy, systematic review, meta-analysis, immunogenicity, clinical trials, durability, dose–response, malaria control

## Abstract

Background: R21/Matrix-M is a pre-erythrocytic malaria vaccine supported by a growing body of field-efficacy, immunogenicity, safety, controlled-challenge, and implementation evidence. We synthesized the contemporary human evidence and quantified protection against naturally acquired clinical malaria. Methods: This systematic review and meta-analysis was conducted and reported in accordance with PRISMA 2020. PubMed, Scopus, Embase, Web of Science, and Cochrane CENTRAL were searched for studies published from January 2021 to February 2026. The review was not prospectively registered, and no separate protocol was prepared. Eleven studies were retained for the systematic review. The primary quantitative synthesis pooled 12-month Cox proportional-hazards estimates for the first episode of clinical malaria from two independent randomized field trials using generic inverse variance methods. Robustness was examined using a fixed-effect model, a sensitivity analysis that disaggregated the phase III seasonal and standard/perennial transmission strata, and a phase III-only analysis. Booster follow-up and controlled human malaria infection (CHMI) studies were summarized descriptively to avoid double counting and mixing of incompatible endpoints. Results: The primary trial-level meta-analysis yielded a pooled hazard ratio (HR) of 0.265 (95% CI 0.235–0.299), equivalent to vaccine efficacy (VE) of 73.5% (95% CI 70.1–76.5%), with no detected heterogeneity (I^2^ = 0%; Q = 0.67, *p* = 0.41; tau^2^ = 0). Disaggregating the phase III transmission strata produced a similar random-effects estimate (HR 0.270, 95% CI 0.223–0.326; VE 73.0%, 95% CI 67.4–77.7%; I^2^ = 53.8%). The fixed-effect estimate was HR 0.270 (95% CI 0.240–0.304), and the phase III-only sensitivity analysis remained strongly protective (HR 0.281, 95% CI 0.220–0.357; VE 71.9%). Booster follow-up supported maintained protection, including approximately 80% efficacy during the 12 months after boosting with the 50-microgram Matrix-M regimen. Across the broader evidence base, the vaccine was generally well tolerated and elicited strong anti-CSP/anti-NANP antibody responses. Conclusions: R21/Matrix-M provides substantial protection against naturally acquired clinical malaria, and the central efficacy estimate is robust to alternative analytic specifications. The quantitative evidence base remains small, however, and longer-term effectiveness, severe-malaria protection, booster timing, and post-deployment safety require continued evaluation.

## 1. Introduction

Malaria poses a grave threat to public health globally, the most recent outbreaks of which took place during 2024, causing 610,000 deaths and a total of 282 million cases, the majority of which occurred in the sub-Saharan region of Africa, mostly affecting children younger than five years of age [1,2,3]. Although gains have been made with respect to the control of vectors, chemoprophylaxis, case management, and intermittent preventive treatment, the WHO Global Technical Strategy has already set its targets for 2020 and 2025 [4]. In this regard, the creation and the implementation of an effective vaccine to counter the predominant parasite, *Plasmodium falciparum*, is generally viewed as a valuable addition to the malaria-control system [5,6]. Thus, the development of R21/Matrix-M (R21/MM), a novel subunit vaccine, can be seen as an advancement in the search for a high-efficacy and scalable malaria vaccine [7].

The R21/MM is a circumsporozoite-based protein vaccine that was created by the collaboration of both the Jenner Institute at the University of Oxford and Serum Institute of India [8], utilizing the Novavax Matrix-M adjuvant to induce immunogenicity [9]. Its design was based on the experience of previous vaccine candidate RTS, S/AS01, which had an efficacy of about 56% over a 12-month period in African children but was unable to meet the WHO efficacy target of 75% [10]. This target was the one that the R21/MM candidate aimed to achieve by optimization of antigen dose, adjuvant level, and synchronization with high-transmission seasons, thereby providing hope for better protective performance and a greater impact on public health [7].

R21/MM clinical trial data have demonstrated promising results. The vaccine efficacy in the low-adjuvant and high-adjuvant arms of the phase 2b trial of the vaccine in Burkina Faso (children aged between 5 and 17 months old) was 74% and 77%, respectively, in the low-dose group after three doses (at 0, 1, and 2 months old) plus a booster dose (at 12 months old) [11]. Vaccine efficacy correlated positively with anti-NANP antibody titres. The phase 3 trial, which enrolled 4878 children in four African countries with both perennial and seasonal transmission (Burkina Faso, Mali, Kenya, and Tanzania), was conducted more recently, with 12-month and seasonal efficacy of 75% and 68%, respectively, in affected regions; furthermore, a booster dose restored efficacy of the vaccine to an approximation of 74% at 18 months in the seasonal setting [12,13]. The findings conclude that the vaccine meets the 75% efficacy target against malaria for at least one year of follow-up, as proposed by the WHO Malaria Vaccine Technology Roadmap. Additionally, the risk profile was favourable, as there were no deaths attributed to the vaccines, and the vaccinated and control groups showed similar data on serious adverse events [14].

Considering such data, a policy recommendation on R21/MM was issued by the WHO in October 2023, recognizing its high efficacy, with administration preceding the high-transmission season; positive cost efficacy, at 2–4 US dollars per dose; and positive benefit–risk balance, with moderate levels of certainty of safety events due to limited availability of data on severe malaria events [15,16]. Public-health impact potential is therefore great: modelling predicts that with extensive deployment in endemic countries, hundreds of thousands of cases would be prevented and the contribution in terms of meeting malaria mortality reduction targets would be substantial [17]. Nevertheless, several critical aspects are still prevalent: the sustainability of protection (longer than 18 months), high-transmission perennial settings, possible interactions with other malaria interventions (e.g., seasonal chemoprevention), logistical and supply-chain issues, and comparative performance relative to RTS, S/AS01 in actual programs [18].

Against this background, our systematic review endeavours to bring together all existing data on the effectiveness (efficacy, immunogenicity, and safety) and response (clinical-endpoint outcomes, multiple-episode incidence, and antibody correlates) of R21/MM on malaria volunteers—namely, children in endemic areas. We evaluate the trial methodology, heterogeneity in the setting of transmission, and duration of follow-up; critically compare the results of R21/MM with those of other vaccine platforms; and consider the factors of real-world implementation and research gaps. Through a strong evidence synthesis, the review aims to guide policy makers, implementation scientists, and malaria-control programs on the strengths and weaknesses of R21/MM and be used to help make strategic decisions on its use in integrated malaria-controlling systems.

## 2. Materials and Methods

### 2.1. Study Design

**Figure 1:**
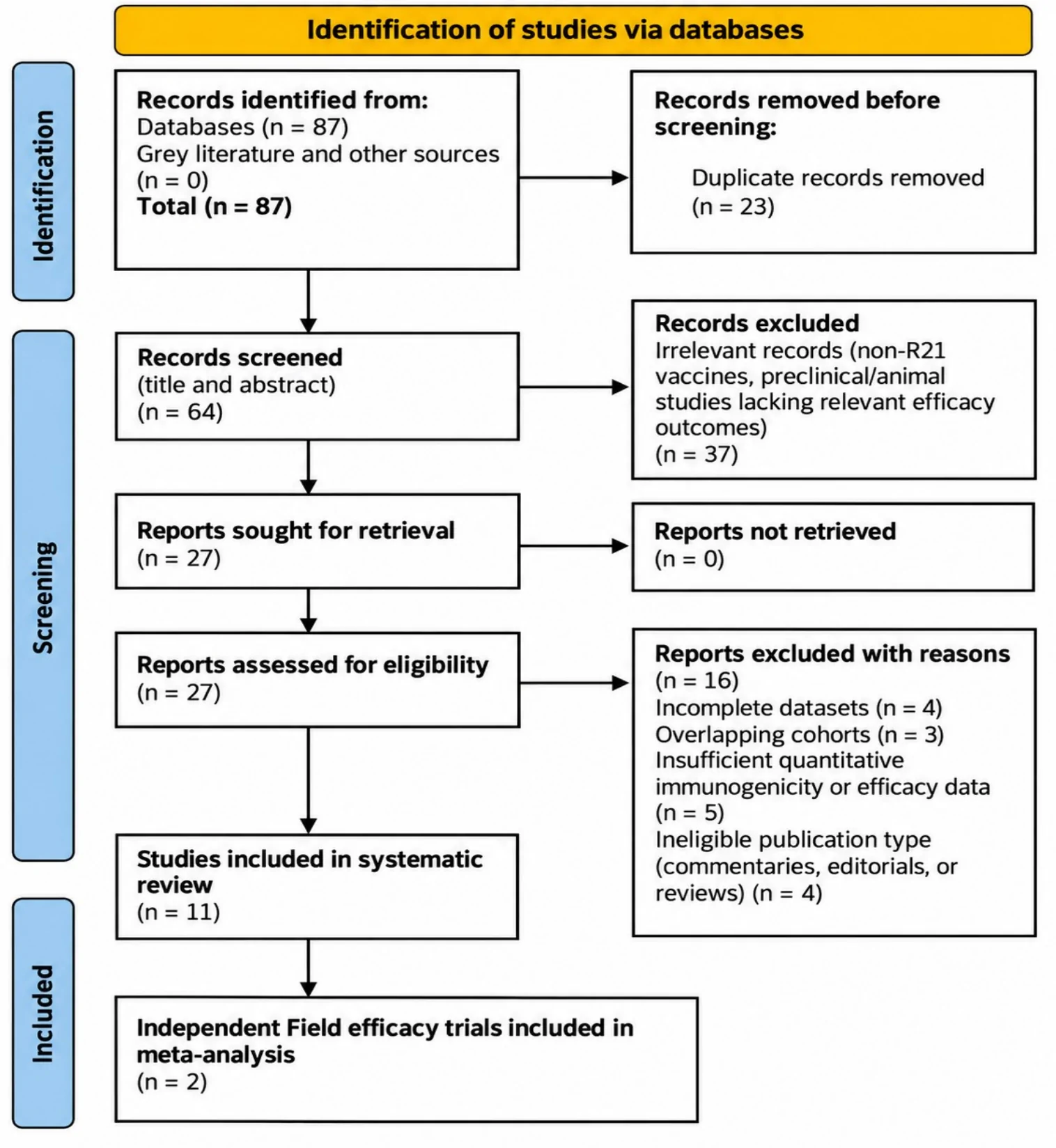
PRISMA flow diagram of study identification, screening, eligibility assessment, and inclusion of the screening process.

This systematic review and meta-analysis was conducted and reported in accordance with the Preferred Reporting Items for Systematic Reviews and Meta-Analyses (PRISMA 2020) statement [19]. The completed PRISMA 2020 checklist is provided in Supplementary Table S1, and the study-selection process is presented in Figure 1. The review was not prospectively registered, and no separate review protocol was prepared; consequently, there were no protocol amendments to report. The review synthesized empirical human evidence on the efficacy, safety, immunogenicity, durability, controlled-challenge response, and implementation-related outcomes of the R21/Matrix-M (R21/MM) malaria vaccine. Randomized controlled trials (RCTs) and eligible observational or mechanistic human studies were considered according to the prespecified eligibility criteria below.

### 2.2. Search Strategy

PubMed, Scopus, Embase, Web of Science, and the Cochrane Central Register of Controlled Trials (CENTRAL) were searched for eligible reports published from January 2021 through February 2026; all electronic sources were last searched in February 2026. WHO publications and the institutional websites/publications of the Jenner Institute and the Serum Institute of India were additionally consulted for emerging R21/MM evidence. These supplementary sources yielded no additional eligible records for inclusion, consistent with the PRISMA flow diagram. Search concepts combined controlled vocabulary, where available, with free-text terms for R21, R21/Matrix-M, Matrix-M, malaria, *Plasmodium falciparum*, vaccine efficacy, immunogenicity, safety, antibodies, and clinical trials. Boolean operators (AND/OR), truncation, and database-appropriate field syntax were used. The database-specific strategies and date limits are reported in Supplementary Table S2.

### 2.3. Inclusion and Exclusion Criteria

Studies were eligible if they (1) were published from 2021 onward [13]; (2) involved human participants, including endemic populations and controlled human malaria infection studies conducted outside endemic settings; (3) evaluated R21/Matrix-M efficacy, safety, immunogenicity, durability, controlled-challenge protection, or implementation-related outcomes; and (4) used randomized, quasi-experimental, observational, mechanistic, or qualitative designs relevant to the review objectives. Studies were excluded if they evaluated non-R21 vaccines without R21/MM data, were preclinical or animal studies, lacked relevant quantitative or qualitative R21/MM outcomes, were commentaries/editorials/narrative reviews, or were published before 2021. Incomplete datasets and overlapping reports were excluded from quantitative pooling when they duplicated participants or outcomes already represented by an included report. For synthesis, studies were grouped as: (i) independent field-efficacy RCTs with comparable 12-month time-to-first-clinical-malaria hazard-ratio estimates for meta-analysis; (ii) booster-extension and controlled human malaria infection studies summarized separately to avoid participant double counting or incompatible endpoints; and (iii) immunogenicity, safety, mechanistic, and implementation studies synthesized narratively.

### 2.4. Screening Process

Study selection followed the PRISMA 2020 process. Eighty-seven records were identified from the searched sources. After removal of 23 duplicates, 64 unique records underwent title/abstract screening by two reviewers working independently. Thirty-seven records were excluded at this stage because they concerned non-R21 vaccines or preclinical/animal work or did not report outcomes relevant to the review question. Twenty-seven full-text reports were sought, and all were retrieved and assessed independently by the same two reviewers. Sixteen full-text reports were excluded because of incomplete datasets (*n* = 4), overlapping cohorts *(n* = 3), insufficient quantitative immunogenicity or efficacy data (*n* = 5), or ineligible publication type *(n* = 4). Disagreements at any stage were resolved by discussion and consensus, with arbitration by a third reviewer when required. Eleven studies met the eligibility criteria and were included in the systematic review. Five reports contributed quantitative efficacy endpoint data to the broader synthesis; only two independent field-efficacy randomized trials met the prespecified criteria for the primary meta-analysis. Challenge studies and the booster extension of the Nanoro cohort were retained for descriptive or mechanistic synthesis rather than entered as independent pooled estimates. The complete study-selection pathway is shown in Figure 1.

### 2.5. Data Extraction and Quality Assessment

Data were collected using a structured extraction form. Two reviewers extracted data independently and resolved discrepancies by consensus. Sought outcomes included first clinical malaria episode/time-to-event efficacy, recurrent malaria, severe malaria, vaccine efficacy and hazard ratios with confidence intervals, safety and adverse events, anti-CSP/anti-NANP antibody responses, durability and booster response, controlled-challenge protection, and implementation or acceptability outcomes. All eligible outcome time points were considered for the narrative synthesis, whereas the prespecified 12-month time-to-first-clinical-malaria endpoint was used for the primary meta-analysis to maximize comparability across independent field trials. Other extracted variables included authorship, publication year, country/site, study design, participant age and sample size, malaria-transmission setting, vaccine dose and schedule, comparator, follow-up duration, randomization and masking methods, attrition, trial registration, and funding source. Methodological quality for the pooled randomized field trials was assessed using the Cochrane Risk of Bias 2 (RoB 2) tool.

### 2.6. Data Analysis

Due to heterogeneity in study designs, transmission settings, participant populations, and performance metrics, the overall evidence base was synthesized narratively and thematically. Study characteristics and risk-of-bias judgments were tabulated, quantitative field-efficacy data were summarized in structured tables, and meta-analytic results were displayed as forest plots. A separate quantitative synthesis was conducted only for independent field-efficacy randomized trials sharing the 12-month time-to-first-clinical-malaria endpoint, as detailed in Section 2.8. Quantitative calculations and forest plots were generated in Python 3.13.5 (Python Software Foundation, Wilmington, DE, USA; https://www.python.org/) using generic inverse-variance calculations based on log-transformed hazard ratios and their standard errors.

### 2.7. Risk of Bias Assessment via ROB-2 Tool

Risk of bias for the two independent field-efficacy randomized trials included in the primary meta-analysis was assessed with the Cochrane RoB 2 framework across five domains: bias arising from the randomization process, deviations from intended interventions, missing outcome data, measurement of the outcome, and selection of the reported result [20]. Judgments were informed by the published trial methods, masking, attrition, endpoint ascertainment, trial registration, and concordance between prespecified and reported outcomes. RoB 2 judgments were reviewed by two reviewers, and disagreements were resolved by consensus. RoB 2 was not applied to qualitative, secondary mechanistic, or non-randomized reports because it is specific to randomized trials; limitations of those designs were considered in the narrative synthesis.

### 2.8. Quantitative Synthesis of Field-Efficacy Data

The quantitative meta-analysis was restricted to independent randomized controlled field trials evaluating R21/Matrix-M against naturally acquired clinical malaria. Controlled human malaria infection (CHMI) studies were not pooled with field-efficacy trials because they involved experimentally administered sporozoites, short follow-up periods, different participant populations, and parasitological or treatment-based endpoints rather than naturally occurring clinical malaria. The 2022 booster publication represented continued follow-up of the same Nanoro phase 2b cohort and was therefore excluded from the primary pooled estimate to prevent double counting [21].

For comparability, the primary meta-analysis used the 12-month time-to-first-clinical-malaria endpoint after the primary vaccination series. In the phase 2b trial, the 5 microgram R21 plus 50 microgram Matrix-M arm was selected because this formulation corresponds to the regimen subsequently evaluated in the phase III trial. Vaccine efficacy in the source trials was defined as VE = 1 − HR, where HR is the hazard ratio from Cox proportional-hazards regression. Reported HRs and 95% confidence intervals were transformed to the natural-log scale, and standard errors were derived from the confidence limits.

Log hazard ratios were pooled using generic inverse-variance methods under a random-effects framework. A fixed-effect model was additionally fitted as a model-specification sensitivity analysis. Between-study heterogeneity was assessed using Cochran’s Q, the I^2^ statistic, and the between-study variance (tau^2^). Because only two independent field-efficacy trials were available, heterogeneity estimates were interpreted cautiously. Funnel plots and regression-based tests for small-study or publication bias were not performed because the number of independent studies was too small for meaningful assessment. No formal GRADE certainty-of-evidence rating was undertaken; confidence in the pooled efficacy outcome was interpreted in relation to randomized study design, RoB 2 judgments, precision, consistency, directness, and the limited number of independent trials.

**Figure 2:**
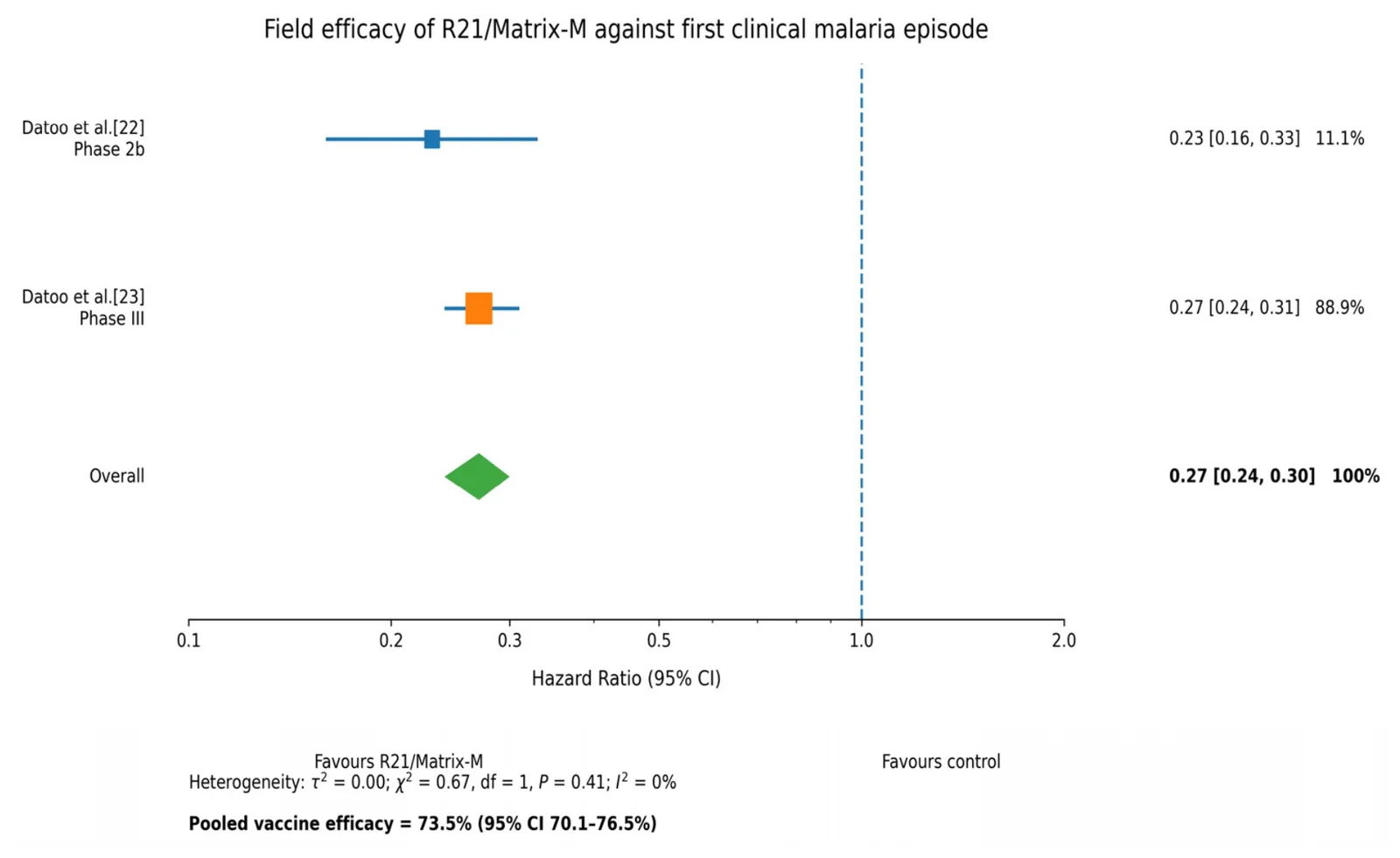
Meta-analysis of field efficacy of R21/Matrix-M against the first episode of clinical malaria. Hazard ratios from two independent randomized controlled field-efficacy trials were pooled using generic inverse-variance methods. Values below HR = 1 favour R21/Matrix-M. The pooled HR was 0.265 (95% CI 0.235–0.299), corresponding to a pooled vaccine efficacy of 73.5% (95% CI 70.1–76.5%). No statistical heterogeneity was detected (I^2^ = 0%, Q = 0.67, *p* = 0.41; tau^2^ = 0). The 2022 booster follow-up and CHMI studies were not included in the pooled field-efficacy estimate. Data are from the two independent field-efficacy trials [22,23]. Squares indicate study-specific HRs, horizontal lines indicate 95% CIs, the diamond indicates the pooled HR, and the dashed vertical line marks HR = 1.

**Figure 3:**
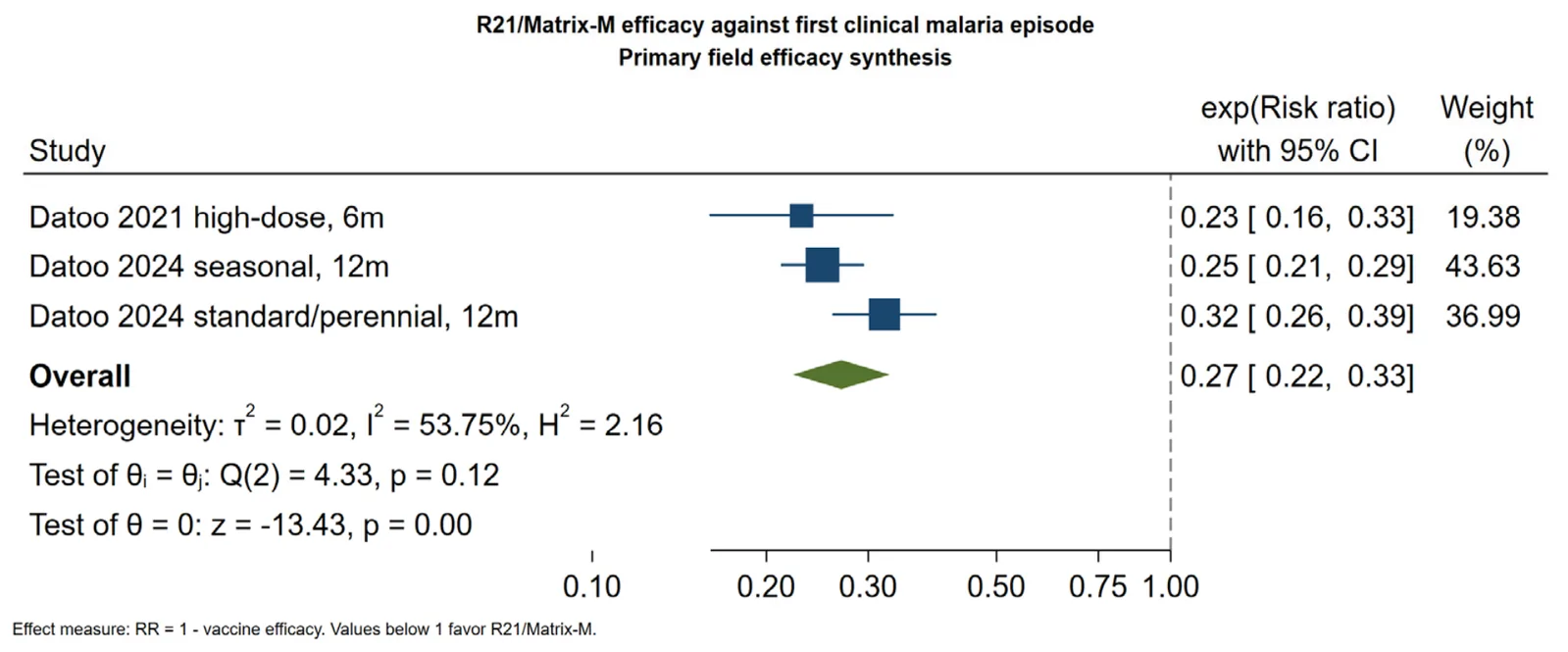
Transmission-strata sensitivity analysis of R21/Matrix-M efficacy against the first episode of clinical malaria. The overall Datoo et al. [22] phase III estimate was replaced by the two non-overlapping seasonal and standard/perennial strata and was not entered simultaneously. The random-effects pooled HR was 0.270 (95% CI 0.223–0.326), corresponding to VE 73.0% (95% CI 67.4–77.7%); I^2^ = 53.8%.

**Figure 4:**
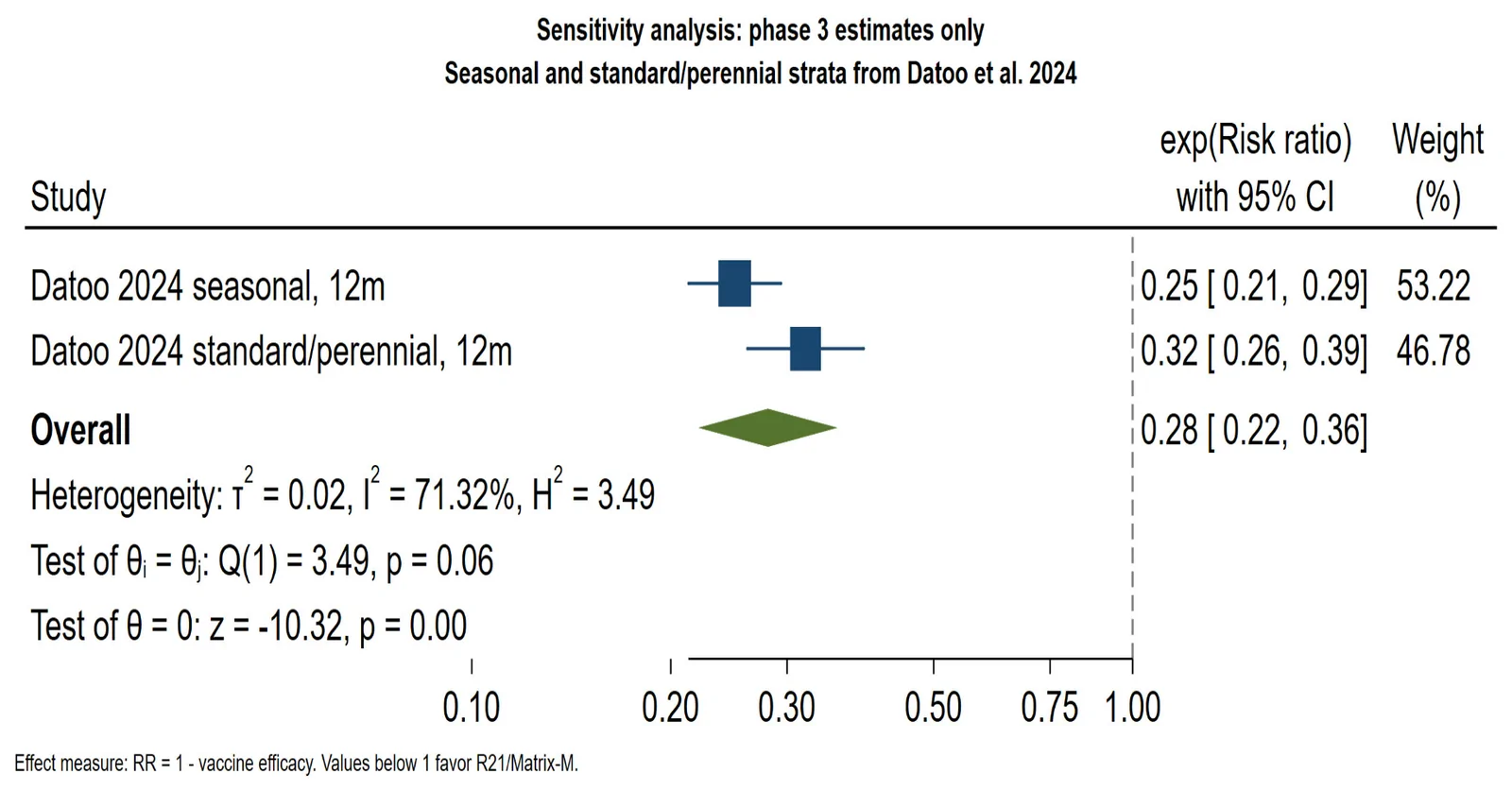
Phase III-only sensitivity analysis using the non-overlapping Datoo et al. [22] seasonal and standard/perennial estimates. The Datoo et al. [23] phase 2b estimate was excluded. The random-effects pooled HR was 0.281 (95% CI 0.220–0.357), corresponding to VE 71.9% (95% CI 64.3–78.0%).

Two additional sensitivity analyses addressed phase III transmission settings. First, the single overall phase III estimate was replaced by the two reported non-overlapping seasonal and standard/perennial strata; the overall phase III estimate and its component strata were never entered in the same model, thereby avoiding participant double counting [22]. Second, a phase III-only analysis pooled only these two non-overlapping strata after removal of the phase 2b estimate [23]. Primary and sensitivity forest plots are shown in Figure 2, Figure 3 and Figure 4.

## 3. Results

### 3.1. Quality Outcome

Eleven studies with diverse designs were included in the systematic review; formal RoB 2 assessment was restricted to the two independent randomized field-efficacy trials contributing to the primary meta-analysis. Randomization processes were consistently well-documented, often using computer-generated sequences and central allocation to prevent selection bias. No substantial deviations from intended interventions were identified, as most trials-maintained blinding of both participants and investigators. Missing outcome data were minimal (<5%) across all studies, and outcome measurements (PCR-confirmed parasitaemia, anti-NANP antibody titres, or clinical malaria episodes) were objectively verified through validated laboratory methods. Reporting bias was considered minimal because all studies were prospectively registered in recognized clinical trial registries, and published reports aligned with pre-specified outcomes. The comprehensive methodological precision increases the credibility and generalizability of outcomes regarding R21/MM vaccine efficacy and immunological response.

### 3.2. Thematic Analysis and Narrative Synthesis

#### 3.2.1. Theme 1: Sustained Efficacy and Transmission-Reducing Potential of R21/Matrix-M Vaccine

The results of Datoo et al. [23] confirm that R21/Matrix-M induces robust immune responses and greater protection than the control, but the effectiveness of the two adjuvant doses should be carefully interpreted. Clinical malaria at the six-month primary endpoint was 29% (43/146) for group 1 (5 μg R21 + 25 μg MM) and 26% (38/146) for group 2 (5 μg R21 + 50 μg MM), drastically different than 71% for the rabies-vaccine control group (105/147). Although these effect sizes may seem impressive, the differences between 74% and 77% efficacy indicate that the higher the dose of Matrix-M, the less significant the increase in the malaria incidence, although there should have been dose-enhanced protection. Protection durability is also indicated by the sustained efficacy of 77% at one year in the 50 MM group (95% CI: 67–84), which cannot be simply compared to that of the 25 MM group because of the lack of one-year efficacy data in the latter [23].

Likewise, Datoo et al. [21] have demonstrated that a high-dose Matrix-M regimen is significantly stronger and longer-lasting compared to a low-dose regimen, although the extent and pattern of this advantage need to be carefully elucidated. At 12 months post booster, vaccine efficacy (VE) in the group immunized with a high dose (5 μg R21 + 50 μg MM) was 80% (95% CI 7285) against the first episode of clinical malaria, followed by 75% through the entire 24 months of the study period following the first three doses. In comparison, the group with a low dose of adjuvant (5 µg R21 + 25 µg MM) indicated a lower VE of 70% (95% CI 5978) at 24 months, and this difference was statistically significant (*p* < 0.0001), showing that a high level of adjuvant concentration provides a significant clinical benefit. In addition, recurrent malaria was covered with 78% (95% CI 7183) against repeated incidences and prevented 2285 incidences of malaria per 1000 child-years at risk in the second year of follow-up with the high-dose group [21].

Similarly, Datoo et al. [22] also showed that the R21/Matrix-M malaria vaccine had an overwhelming 73% efficacy (95%) in clinical malaria among children aged 5 to 36 months during a 12-month follow-up, representing a significant step in terms of countering malaria in vulnerable groups. The overall similar performance of the vaccine in both seasonal (75%) and standard (68%) environments with transmission also underscores its capability to adapt to a wide diversity of epidemiological arrangements, which enhances its possibility of universal use in endemic areas. More importantly, the protective effect was evident within a short interval after immunization and lasted during the course of observation, even though there was a progressive reduction in the antibody titres, implying the development of long-term immunological memory. Furthermore, the cross-sectional parasitological data, with significantly lower rates of parasite prevalence of 2.5–2.0 at ages of 12 and 18 months, respectively, compared with prevalence rates of 5.1–4.4 in the control group (*p* < 0.0001 and *p* = 0.0014), were very convincing as an indication of the vaccine’s ability to counter and prevent clinical attacks and to interfere with the dynamics of transmission at the community level [22].

Meanwhile, Venkatraman, Silman, et al. [24] showed that although several dosing regimens of R21/Matrix-M provide significant protection, the dose most effective in all cases is the standard three-dose scheme of 10 μg R21 with 50 μg Matrix-M monthly, which resulted in 9/11 subjects being uninfected (~80%). The 50/50/10 μg and 10/10/2 μg schedules (fractional third dose) generated protective efficacies of about 63% and 77% respectively, although the studies reported no statistical difference between these schedules and the usual one, indicating an overlapping confidence interval and possibly equal efficacy under real-world conditions. The results of the two-dose schedule were approximately 65%, with a cumulative improvement in efficacy with a higher number of doses, but the contribution value of the entire three-dose schedule should be counterbalanced with the cost of running the operation [24].

#### 3.2.2. Theme 2: Safety Profile and Limitations in Severe Malaria Protection

Regarding safety, Datoo et al. [22] noted that R21/Matrix-M was well tolerated overall, with mild and moderate events reported seven days after immunization. This supports the effective reactogenicity profile of the vaccine in large-scale use in children. Notably, severe malaria and other adverse incidents were rare, with only 12 cases occurring in a 12-month period, including five events at seasonal sites and seven events at traditional sites. Despite the estimated high vaccine efficacy of 67% (95% CI −4 to 90; *p* = 0.058), the confidence interval was large, and there was no statistical significance, indicating that the study was underpowered to prove protection against the most life-threatening forms of the disease. The 18-month investigation yielded an efficacy profile of 65% (*p* = 0.073), suggesting a promising trend but leaving a question mark due to the small sample size and the infrequency of events.

Likewise, Hanboonkunupakarn et al. [25] also confirmed the validity of R21/Matrix-M when used concomitantly with antimalarials, but some trends cannot be interpreted without caution in terms of tolerability. The proportions of events solicited as local and systemic adverse events were similar in the vaccine-plus-antimalarials group (74%, 37/50) and the vaccine-only group (72%, 36/50), and none of the events was reported in the no-vaccine control group (Group 3), which confirms the presence of vaccine reactogenicity and not background symptoms. Unsolicited adverse events were less common in the combination group (34%, 17/50) compared to the vaccine-only group (12%, 6/50), but none was severe or due to the interaction between vaccines and drugs, however, the difference in frequency is questionable with respect to whether additive, non-causal symptom burdens may continue to play a role in acceptability when used at scale. There was just one severe adverse event that was observed in the whole cohort and was considered to have no relationship with vaccination, which supports the positive safety profile [25].

Besides that, Sang et al. [26] provided strong reasons to believe in the safety of the R21/Matrix-M vaccine. Although the adverse events were mainly mild and self-limiting, reminiscent of other pre-erythrocytic malaria vaccines, such as local injection-site reactions and transient systemic reactions, the study was limited in its capacity to identify rare or delayed adverse events due to its size and follow-up period. The lack of vaccine-associated serious adverse events (SAEs) is encouraging, especially among infants and young children, which are the major target population, yet the active use of passive reporting and relatively short surveillance periods may underestimate rare, infrequent immunological events or long-term reactogenicity patterns [26].

Meanwhile, in a recent trial, Venkatraman, Tiono, et al. [27] also emphasized that the reactogenicity profile of R21/Matrix-M is significantly less than that of the standard RTS, S vaccine; however, the size and nature of that difference present significant implications regarding the broader deployment. The significant safety benefit of UK participants reporting no vaccine-related fever following the administration of the 50 μg doses of R21/Matrix-M is important, as RTS, S tends to induce febrile responses, which makes it difficult to monitor post vaccination in malaria-infested areas. Interestingly, reactogenicity was even reduced in Burkinabe adults compared to UK adults on the same dose of 10 μg, and this might indicate that the target population, which is the adult population in endemic regions, is more suitable for vaccination [27].

In line with this, Kapulu et al. [28] provided route-specific evidence that R21/Matrix-M protection against *P. falciparum* sporozoites is strongly dependent on how parasites enter the host. In this phase 2b controlled human malaria infection study, none of the 12 R21/Matrix-M-vaccinated participants challenged intradermally with PfSPZ met the primary treatment endpoint, indicating complete clinical protection in this route-specific challenge model. In contrast, all five R21/Matrix-M-vaccinated participants challenged by direct venous injection developed typical parasite growth curves and met the primary endpoint, showing that the same vaccine-induced immunity failed when sporozoites were delivered directly into the bloodstream (*p* < 0.0005 by log-rank analysis across groups). This finding was also supported by qPCR outcomes, where 75% of the R21 intradermal group remained PCR-negative and the remaining 25% showed only transient untreated PCR positivity, whereas 100% of the R21 direct-venous group required treatment.

The secondary efficacy outcomes followed the same biological pattern. R21-vaccinated volunteers receiving intradermal challenge did not meet any secondary parasitaemia endpoints, while participants in the control, ME-TRAP and R21 direct-venous groups reached parasitaemia thresholds of >20, >500, >1000 and >10,000 parasites/µL between days 7 and 21, with statistically significant differences across groups. Although anti-NANP antibody responses increased strongly after R21 vaccination, rising from baseline levels below 10 ELISA units to above 1000 ELISA units, protection was only observed against intradermal challenge. Therefore, the study suggests that anti-CSP antibodies generated by R21/Matrix-M can efficiently neutralise dermal sporozoites but may be insufficient against venous sporozoites, supporting the idea that breakthrough malaria after CSP-based vaccination may occur when mosquito bites occasionally deposit parasites directly into capillaries [28].

Mechanistically, this route-dependent protection is important because it helps explain why high anti-CSP antibody titres do not always translate into uniform protection in field settings. Kapulu et al. [28] argued that infectious mosquito bites deliver a mixture of dermal and venous sporozoites and that dermal sporozoites are more readily blocked by antibodies than venous sporozoites. The complete separation of outcomes between the R21 intradermal group and the R21 direct-venous group, despite the small sample size, therefore supports the need to evaluate antibody-based malaria vaccines using separate intradermal and venous challenge models. This approach may allow for more accurate identification of protective antibody thresholds and may be particularly useful for comparing next-generation CSP-based vaccines with higher-potency antibody responses.

#### 3.2.3. Theme 3: Dose-Dependent Immunogenicity Strengthening Humoral Correlates of Protection

The high efficacy is mechanistically explained by Datoo et al. [23] but also exposes the important dose–response dynamics that should be critically considered. R21/Matrix-M caused a strong antibody response against the CSP NANP repeat region, but the intensity of this response varied significantly between groups: children vaccinated with the 50 ug dose of Matrix-M generated antibody titres that were 86% greater than those generated by the children vaccinated with the 25 ug dose of Matrix-M, indicating a strong immunological benefit of the higher adjuvant concentration. The transient nature of primary humoral immunity is demonstrated by the fact that ELISA-measured levels of antibody peaked 28 days following the third dose, after which the levels declined during the following months. The fourth and final dose at one-year restored titres to levels similar to those following the post-dose-three peak, indicating good immune memory [23].

Likewise, the immunogenicity results presented by Sang et al. [26] show an exceptionally high level of antibody response to CSP NANP repeats, although the results need a critical approach to dose optimization and long-term stability. The drastically rising geometric mean titres, which start at 1.1 EU/mL, which is equivalent to 1.1 EU/mL, and reach 10,175 EU/mL at day 84 in infants, may indicate strong early immunogenicity though the heavy use of quantitative IgG titres but not the parallel measure of functional antibody activity, including complement fixation or sporozoite inhibition, restricting the interpretation of how these titres are translated into protective efficacy. Although one-way ANOVA and Tukey post hoc tests prove the statistical superiority of the titres of the higher-dose groups, the research does not entirely detail whether the quantitative difference can generate a meaningful clinical benefit or whether it is due to a mere transient immunological stimulation. The reported declines in the levels of antibodies as time goes on and eventual recovery upon a booster dose (GMT 6792 EU/mL at day 456) only further complicate the issue of obtaining lasting immunity in infants and raise the question of whether an annual booster may be necessary to sustain protection [26].

On the same note, Datoo et al. [21] presented strong evidence that anti-NANP antibody titres are significant and measurable measures of protection; however, the magnitude of these associations suggests that the intensity of antibodies may not comprehensively explain the protective immunity. The antibody levels 28 days following the last vaccination had a significant association with clinical protection in the high-dose Matrix-M group, with Spearman correlation coefficients of −0.32 (*p* = 0.0001) in the first year and −0.20 (*p* = 0.02) in the second year, with better antibody levels being strongly associated with a lower risk of malaria. It was interesting to note that the participants who surpassed these in the high-dose group had a 77% in risk of malaria, which indicates that there is a definite dose–response relation of humoral immunity [21]. Thus, these results support the significance of high antibody titres, accompanied by a need to conduct further mechanistic studies to establish a complete set of correlates of vaccine-induced protection.

Conversely, Bundi et al. [29] emphasized an age-related trend in response to the R21/Matrix-M vaccination, which is rather complex, or divergent age-related patterns and raised important questions about the role of age in determining the extent and duration of immunity caused by vaccines. Although infants and children elicited significantly better early increases in IgG levels with all the tested antigens (full-length R21, NANP repeats, C-terminus of CSP, and HBsAg), the persistence of these increases past one year was significantly worse in children than adults, despite a similar substantial increment in the post-vaccination increases (*p* < 0.0001). Adults, in their turn, produced quantitatively lower peak responses and showed better antibody persistence following the primary series in terms of percentages of median durability of anti-full-length R21 (17.3% vs. 6.7% in children), anti-NANP (38.3% vs. 6.9%), and anti-C-terminus antibodies (24.8% vs. 5.0%). This divergence suggests that adult immune systems may have prioritized long-term memory development over short-term effector development, whereas younger immune systems disproportionately support high-magnitude but short-lived anti-infection responses.

Furthermore, as Bundi et al. [29] found, the change in the dose of the antigen (5 µg vs. 10 µg) in infants did not result in statistically significant differences in the quantitative antibody titres; thus, it is possible that the antigen amount is not the leading cause of humoral magnitude in infants. Nevertheless, the adjuvant dose (50 µg Matrix-M) used in the higher dose greatly improved functional immune markers, including sporozoite invasion inhibition and C1q fixation, which supports the ultimate role of adjuvant potency in developing functional immunity, not merely antibody levels. Although the close associations of IgG levels and functional assay results support the argument that the quality of the antibodies mediates vaccine efficacy, the study itself does not give much mechanistic insight as to why adjuvant escalation enhanced functionality but did not raise titres [29].

#### 3.2.4. Theme 4: Community Acceptance Driven by Perceived Efficacy and Health Benefits

The high malaria burden in the study area was a pivotal factor in the acceptability of the R21/Matrix-M malaria vaccine, which created a desperate local need for preventive measures [30]. The favourable societal attitudes were largely due to the perceived high effectiveness of the vaccine, as participants noted visibly healthier children and quicker recovery in the vaccinated group during the trial. This practical data, supported by the solid efficacy results of the Phase III trial, created a sense of confidence and optimism in the vaccination process. The paper also observed that receptivity increased with trust in the trial team and past positive experiences with health research, which demonstrated that social capital and historical participation are important determinants of vaccine acceptance among the population. An important inference that can be drawn is that, due to the perceived superiority of the vaccine, some caregivers overvalued other preventive strategies, especially seasonal malaria chemoprevention (SMC), indicating a possible danger of complacency in the full control of malaria.

Another important element of the outstanding contribution of trust and confidence to clinical research is the key factor in influencing the overall reaction of the community to the R21/Matrix-M vaccine [30]. The participants appreciated the quality and free healthcare services offered in the course of the trial, which helped to build good will and strengthen the sense of security and legality. However, the study also found some gaps in the knowledge of clinical trials, such as randomizing and the use of a placebo, implying that the informed consent, though ethically non-threatening, may not have been converted into a full understanding of the trial design by all the participants. This leads to significant ethical and communication-related concerns, and the lack of understanding of these fundamental notions may lead to the manipulation of expectations and attitudes towards post-trial vaccinations.

Meanwhile, the IgG response was also remarkably similar and high-level, and the surprising lack of group-to-group differences across all R21/Matrix-M vaccine antigens is also telling about the immunological consequences of antimalarial co-administration. Vaccine-plus-antimalarials (Group 1) and vaccine-only (Group 2) groups had nearly complete seropositivity on the NANP6, C-terminal, and full-length R21 antigens, with 96–100% seropositivity on NANP6 at 3 months and 96 to 100% seropositivity at 6 months of age against the unvaccinated control group (Group 3), where seropositivity was minimal (10–33%). Geometric mean antibody levels did not differ significantly between the two vaccinated groups because geometric mean ratios were near to one and the 95% confidence intervals were narrow, which showed that antimalarial co-administration induced immunogenicity in the two vaccinated groups that was equal in magnitude to the vaccine alone. Importantly, such absence of divergence in antibody concentrations, even in the hypothetically problematic context of antimalarial pharmacodynamics potentially interfering with antigen presentation or immune activation, indicates that the R21/Matrix-M platform is immunologically strong, even under pharmacologically complicated conditions.

On the other hand, Venkatraman, Tiono, et al. [27] also demonstrated that R21/Matrix-M induces robust antibody responses, even at substantially lower antigen doses, although the comparable titres across doses call for careful scrutiny of dose optimization. Geometric mean IgG titres against NANP measured 28 days post third vaccination were high at both 2 μg and 10 μg antigen doses, implying the potential for dose sparing and improved manufacturing efficacy, yet it remains unclear whether qualitative antibody properties—such as avidity—are truly comparable across these doses. Durable antibody responses were observed in the phase 1a trial at all doses for at least 6 months post final vaccination and in the phase 1b trial at 9 months, specifically for the 10 μg doses, demonstrating promising longevity for a low-dose formulation. Importantly, strong immunogenicity at a 10 μg dose was equivalent to responses seen at 50 μg, and this dose also exhibited superior safety relative to RTS, S, suggesting an advantageous immunogenicity-to-reactogenicity ratio [27].

### 3.3. Risk-of-Bias Assessment via ROB-2

The two independent randomized field-efficacy trials contributing to the primary meta-analysis were judged at low risk of bias overall. Both trials used randomized allocation, blinded or double-blind procedures, objective laboratory-confirmed clinical malaria endpoints, prospectively registered protocols, and prespecified efficacy analyses. Attrition was limited and handled within time-to-event analyses. The broader systematic-review evidence included open-label early-phase studies, CHMI studies, secondary analyses of archived trial samples, and a qualitative acceptability study; these designs were not assigned RoB 2 ratings, and their limitations were considered in the narrative synthesis. Table 1 summarizes RoB 2 judgments for the pooled field trials, and Table 2 summarizes the characteristics of all included studies.

### 3.4. Meta-Analysis of Field Efficacy

Five reports contained quantitative efficacy endpoint data in the systematic review, but only two independent randomized field-efficacy trials provided sufficiently comparable 12-month time-to-first-clinical-malaria data for pooling (Table 3; see Supplementary Table S3 for the detailed data extraction and summary of selected studies). These were the phase 2b trial conducted in Nanoro, Burkina Faso, and the multicentre phase III trial conducted across Burkina Faso, Mali, Kenya, and Tanzania.

In Datoo et al. [23], 39 of 146 children receiving 5 micrograms of R21 plus 50 micrograms of Matrix-M experienced a first episode of clinical malaria during 12 months of follow-up, compared with 106 of 147 children in the rabies-vaccine control group. The reported hazard ratio was 0.23 (95% CI 0.16–0.33), equivalent to 77% vaccine efficacy. In the phase III trial reported by Datoo et al. [22], 464 of 3102 R21/Matrix-M recipients experienced a first clinical malaria episode, compared with 627 of 1541 controls, corresponding to an HR of 0.27 (95% CI 0.24–0.31) and approximately 73% vaccine efficacy.

#### 3.4.1. Pooled Field-Efficacy Estimate and Interpretation

Pooling the two independent trials produced an HR of 0.265 (95% CI 0.235–0.299), corresponding to a pooled vaccine efficacy of 73.5% (95% CI 70.1–76.5%). The phase III trial contributed approximately 88.9% of the inverse-variance weight, and the phase 2b trial contributed 11.1%. No statistical heterogeneity was detected (Q = 0.67, df = 1, *p* = 0.41; I^2^ = 0%; tau^2^ = 0). Because only two trials contributed to the pooled analysis, the absence of detected heterogeneity should not be interpreted as proof of identical effects across all epidemiological settings. The individual trial estimates and the pooled field-efficacy estimate are shown in Figure 2.

The phase III study additionally reported high efficacy in both seasonal and standard/perennial transmission settings (approximately 75% and 68%, respectively). These strata were not entered alongside the overall phase III estimate in the primary meta-analysis. Instead, they were used only in sensitivity analyses, where they replaced the overall Datoo et al. [22] estimate, so no participants were double counted. These site-setting estimates were not treated as independent studies in the meta-analysis because they were subgroups of the same randomized phase III trial.

The 2022 booster-extension publication was also excluded from the pooled estimate because it followed the same phase 2b Nanoro cohort. It was retained as durability evidence: in the 5-microgram R21 plus 50-microgram Matrix-M group, efficacy against the first clinical malaria episode was approximately 80% during the 12 months after the booster and 75% across 24 months after the primary series.

CHMI studies were interpreted separately as mechanistic evidence and were not incorporated into the field-efficacy forest plot. Their experimental exposure, short follow-up, and parasitological endpoints are not directly commensurable with naturally acquired clinical malaria in endemic children.

#### 3.4.2. Sensitivity Analysis

Replacing the phase III overall estimate with its non-overlapping seasonal and standard/perennial strata generated three effect estimates: HR 0.23 (95% CI 0.16–0.33) for phase 2b, HR 0.25 (95% CI 0.21–0.29) for seasonal phase III sites, and HR 0.32 (95% CI 0.26–0.39) for standard/perennial phase III sites. The random-effects pooled HR was 0.270 (95% CI 0.223–0.326), equivalent to VE 73.0% (95% CI 67.4–77.7%). Heterogeneity was moderate (I^2^ = 53.8%; tau^2^ = 0.0151; Q = 4.33, *p* = 0.115), consistent with some setting-specific variation rather than loss of overall protection. This transmission-strata sensitivity analysis is shown in Figure 3.

The fixed-effect analysis of the three disaggregated estimates gave HR 0.270 (95% CI 0.240–0.304), closely matching the primary result. A phase III-only random-effects analysis, restricted to seasonal and standard/perennial strata, yielded HR 0.281 (95% CI 0.220–0.357), equivalent to VE 71.9% (95% CI 64.3–78.0%). Thus, the protective conclusion did not depend on the smaller phase 2b study or on the choice between trial-level and transmission-stratified specifications. The phase III-only sensitivity analysis is shown in Figure 4.

#### 3.4.3. Durability and Booster-Status Analysis

**Figure 5:**
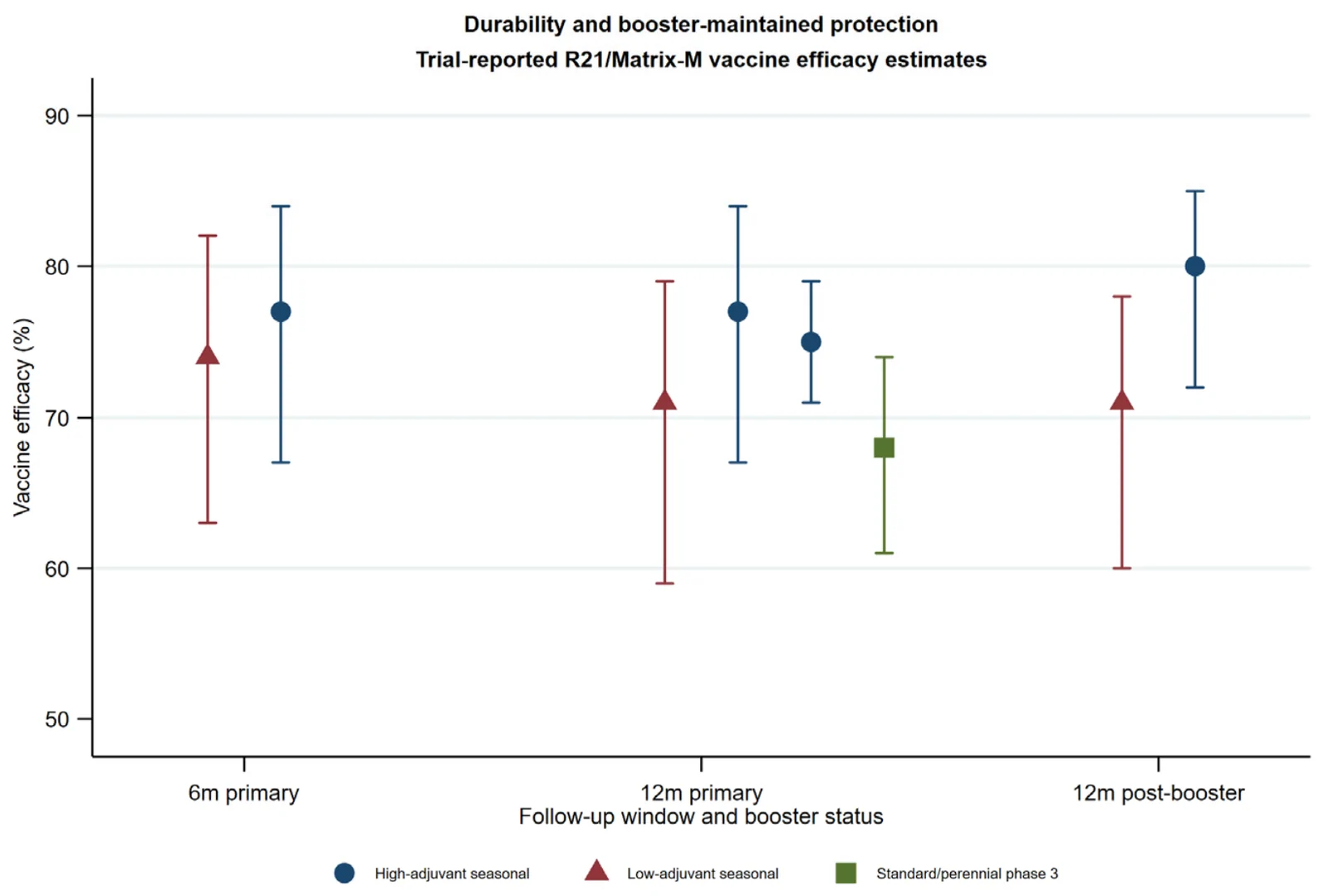
Durability and booster-status profile of R21/Matrix-M efficacy.

Durability was summarized descriptively rather than pooled as a formal subgroup meta-analysis because several estimates were repeated follow-up measurements from the same parent cohort. Figure 5 shows high early efficacy after the primary series and maintained protection after booster dosing. In the Burkina Faso phase 2b trial, 6-month efficacy was 74% in the low-adjuvant arm and 77% in the high-adjuvant arm, and twelve months after the primary series, efficacy was 71% and 77%, respectively. After the 12-month booster, reported efficacy was 71% in the low-adjuvant booster arm and 80% in the high-adjuvant booster arm. The individual field-efficacy estimates and their analytic roles are summarized in Table 4, while the primary and sensitivity meta-analysis results are summarized in Table 5.

Durability was summarized descriptively rather than pooled because repeated follow-up estimates came from the same parent cohort. In the high-adjuvant Nanoro group, reported efficacy was approximately 77% 12 months after the primary series, 80% during the 12 months after the booster, and 75% across 24 months after the original three-dose series [21,23]. These data support booster-maintained protection but should not be interpreted as independent meta-analytic observations.

### 3.5. Reporting Bias and Certainty of Evidence

Formal statistical assessment of small-study effects or publication bias was not undertaken because only two independent field-efficacy trials contributed to the primary meta-analysis. A formal GRADE certainty rating was also not performed. Nevertheless, both pooled studies were randomized controlled trials judged at low risk of bias, their effect estimates were directionally consistent and precise, and the sensitivity analyses produced similar protective estimates. The small number of independent trials remains an important limitation when interpreting heterogeneity, reporting bias, and overall confidence in the evidence base

## 4. Discussion

The results of this systematic review indicate the high immunogenic potential of the R21/Matrix-M vaccine in preventing clinical malaria; a 70–80% range of the efficacy rate was obtained for different dosages and transmission environments [21,22,23,24,27]. These findings support the earlier research that has tested the other malaria vaccines, like RTS, S, which has shown lower efficacy against clinical malaria in some settings and at longer follow-up (approximately 30% or lower in some analyses), with protection that can wane over time [29]. The high observed efficacy of R21/Matrix-M may be partly attributable to its vaccine design, including the high density of circumsporozoite protein (CSP) epitopes on R21 particles and the immunostimulatory properties of the Matrix-M adjuvant. Matrix-M has been shown to enhance both humoral and cellular immune responses in several vaccine platforms, including recombinant protein-based SARS-CoV-2 vaccine NVX-CoV2373 [31,32]. Comparing the results of the efficacy of R21/Matrix-M with those of RTS, S directly, it can be observed that it is a notable leap in the process of malaria vaccine development, as it has not only greater initial efficacy but also a longer course of protection, which is essential in malaria control over time. However, cross-vaccine comparisons should be interpreted cautiously because R21/Matrix-M and RTS, S/AS01 have been evaluated for different populations, transmission settings, dosing schedules, endpoints, and follow-up periods. The evidence-base comparison is summarized in Table 6.

Regarding the long-term efficacy of vaccine protection, the provided data suggest that protection remains effective for up to two years after vaccination, even in malaria-endemic areas [22,27]. This is in contrast to RTS, S, which exhibited a short-lived increase in efficacy with time, especially in children [33]. Research on other malaria vaccines, such as the PfSPZ vaccine, which is also associated with promising long-term immunity, supports the high durability of the R21/Matrix-M vaccine [34]. The prolonged presence of the immune response may be explained by the efficient stimulation of both humoral and cellular immunity, as supported by immune profiling studies. This makes the R21/Matrix-M vaccine a more credible option for malaria eradication programs, particularly in areas with frequent malaria occurrences.

The available durability evidence supports a fourth dose approximately 12 months after the primary series, but it does not yet establish that repeated annual boosting is superior to seasonally timed boosting across all transmission settings. In highly seasonal settings, administering booster doses shortly before the high-transmission period can align the period of strongest protection with peak malaria risk. In perennial settings, particularly those with seasonal peaks, age-based, seasonal, or hybrid delivery approaches may be considered; WHO guidance also allows for consideration of an additional dose one year after dose 4 where substantial malaria risk persists [35]. Programmatically, seasonal boosting may maximize temporal alignment with risk but requires synchronized campaigns, whereas age-based delivery may integrate more easily with routine immunization. Comparative effectiveness and cost-effectiveness studies should therefore evaluate coverage, missed opportunities, delivery costs, vaccine wastage, and local transmission seasonality before one booster strategy is preferred over another.

The field-efficacy meta-analysis strengthens the clinical interpretation of the evidence by restricting pooling to two independent randomized trials that used a comparable 12-month time-to-first-clinical-malaria endpoint. The pooled HR of 0.265 corresponds to approximately 73.5% vaccine efficacy (95% CI 70.1–76.5%), with closely aligned trial-specific estimates despite differences in sample size, geographic coverage, participant age, and transmission pattern. The transmission-strata sensitivity analysis remained concordant with the primary model (HR 0.270, 95% CI 0.223–0.326), while the phase III-only analysis also remained strongly protective (HR 0.281, 95% CI 0.220–0.357). Because the seasonal and standard/perennial strata are non-overlapping and replaced rather than accompanied the overall Datoo et al. [22] estimate, these analyses avoid participant double counting while making transmission-setting variation more visible. The 2022 booster extension was retained as durability evidence rather than an independent study, thereby avoiding double counting of the Nanoro phase 2b cohort. CHMI studies remain valuable for mechanistic interpretation but were not combined with field trials because experimentally induced infection is not directly comparable with naturally acquired clinical malaria.

The heterogeneity findings also have programmatic implications. Although no heterogeneity was detected in the primary two-trial model (I^2^ = 0%), the transmission-strata sensitivity analysis showed moderate heterogeneity (I^2^ = 53.8%; *p* = 0.115), with point efficacy higher in seasonal sites than in standard/perennial sites. The non-significant Q-test and the small number of estimates mean that this variation should be treated as hypothesis-generating rather than as evidence of a definitive effect modifier. Nevertheless, it supports tailoring implementation to local transmission ecology: pre-transmission scheduling may be advantageous where malaria risk is sharply seasonal, whereas perennial settings may require consistent routine delivery, timely booster coverage, and continued integration with vector control and chemoprevention. Different national schedules should not be inferred from these subgroup data alone; prospective comparative-effectiveness studies across transmission ecologies are needed.

Safety of the R21/Matrix-M vaccine, as reported in this review, is also quite positive, and most of the adverse events are mild or moderate in nature, including localized reactions at the site of injection or temporary systemic reactions [22]. This is sharply opposed to other malaria vaccines like RTS, S, which has been linked with increased incidences of febrile reactions, particularly in young children [7]. Moreover, compared to the antimalarial drug regimens, including the artemisinin-based combination therapies (ACTs), which are also associated with resistance development and side effects [9], the R21/Matrix-M vaccine is a more sustainable and preventive solution to the control of malaria, and this may ease the load on frontline therapeutic drugs.

Regarding the applicability of the vaccine in various transmission contexts, the results highlight its generalizability, as its effectiveness has consistently been above 70% in both seasonal and perennial areas of transmission [22]. This flexibility is a major benefit compared to various malaria complications such as seasonal malaria chemoprevention (SMC), which, though effective in some endemic areas, has seasonality and logistical limitations [36]. The R21/Matrix-M offers year-long coverage, which is a huge advancement in the practice of malaria vaccination, particularly when compared to intermittent malaria treatment methods such as SMC. Also, as demonstrated in research done on other vaccine candidates like the PVX/AS01 [37], the efficacy of vaccines tends to change in various epidemiological conditions, which once again emphasizes the importance of the consistency of R21/Matrix-M in different transmission environments.

Considering the dose–response dynamics, the reviewed studies indicate the immunological benefits of increased dosage of the Matrix-M regimen in a positive manner [21,23]. This observation is supported by other studies on vaccines, including a study on the dose-dependent efficacy of the BCG vaccine against tuberculosis [38]. This is a dose-dependent increase in immune activity that has been a recurring motif in vaccination development, implying that adjuvants such as Matrix-M are essential in maximizing vaccine efficacy. The case of Matrix-M being a better adjuvant in the design of malaria vaccines is further reinforced by the fact that R21/Matrix-M showed similar or better clinical protection using lower adjuvant dosages [24]. The subtle picture of the use of adjuvants in R21/Matrix-M, compared to other vaccines such as RTS, S, which exhibited no dose-dependent response, represents a promising outlook for optimization in malaria vaccine trials.

Protection against severe malaria remains an important evidence gap. In the phase III trial, R21/Matrix-M showed an estimated 67% vaccine efficacy against severe malaria (95% CI −4 to 90; *p* = 0.058), but severe-malaria events were uncommon, and the analysis was underpowered [22]. A definitive assessment would require a substantially larger multicentre phase III/IV trial or post-licensure effectiveness study in high-burden settings, with severe malaria specified prospectively as a key endpoint, harmonized case definitions, and sufficiently long follow-up to accrue an adequate number of events. An event-driven design is preferable to quoting a single fixed sample size: the required enrolment should be calculated from the expected severe-malaria incidence in the control group, the vaccine effect that the study aims to detect, allocation ratio, follow-up duration, and anticipated losses to follow-up. A pooled severe-malaria meta-analysis is not currently robust because few independent R21 field trials provide comparable severe-malaria endpoints and the event counts are sparse. As additional trials and longer phase III follow-up become available, harmonized aggregate-data meta-analysis or, preferably, individual-participant-data meta-analysis could improve precision and permit analyses by transmission setting, age, and booster status.

Furthermore, the large-scale implementation of the R21/Matrix-M vaccine is due to the community’s acceptance of the vaccine [30]. The results of this research indicate that the acceptance of vaccines has a significant relationship with perceived efficacy, trust in the clinical research procedure, and the malaria burden in the community. This is similar to the acceptance issues of other malaria vaccines like RTS, S that also experienced doubt and resistance in some endemic areas. The scientific effectiveness of R21/Matrix-M is not the only factor that may determine its utility as a tool to achieve specific health outcomes within a broader preventive health strategy, with possible emphasis on education and community involvement, as well as on ensuring that people have no concerns about vaccine safety. This will be crucial to the overall acceptance and use of the vaccine, especially in places where malaria is causing a public health emergency.

### Limitations

Several limitations qualify the quantitative findings. First, only two independent field-efficacy randomized trials contributed to the primary meta-analysis, limiting statistical power to detect between-study heterogeneity and precluding meaningful assessment of publication bias by funnel plot or regression tests. Although I^2^ was 0%, this should be interpreted cautiously because heterogeneity statistics are imprecise when very few studies are available. Second, the phase III trial contributed most of the statistical weight because of its substantially larger sample size. Third, the trials differed in geographic coverage, participant age, malaria transmission intensity, and timing of vaccination relative to the transmission season. The stratum-based sensitivity analyses should not be interpreted as adding independent trials: the seasonal and standard/perennial estimates are non-overlapping subgroups of the same phase III study and were used only as replacements for its overall estimate. The booster-extension publication represented continued follow-up of the phase 2b cohort and was deliberately excluded from pooling to prevent double counting. Finally, CHMI studies were not included in the field-efficacy meta-analysis because their experimentally administered sporozoite exposure, short follow-up, and parasitological endpoints are not directly equivalent to naturally acquired clinical malaria.

The review process itself also has limitations. The search was restricted to evidence published from 2021 through February 2026, so earlier developmental publications were outside the prespecified eligibility window. Formal certainty grading was not undertaken, and the small number of independent efficacy trials prevented statistical assessment of reporting bias. These methodological constraints should be considered alongside the clinical and statistical limitations of the included evidence

## 5. Conclusions

The R21/Matrix-M shows substantial efficacy against first clinical malaria episodes in available field efficacy studies of children in endemic African settings. The pooled estimate suggests approximately 73% vaccine efficacy, with phase III -only sensitivity analysis supporting a similarly strong protective effect. Descriptive durability evidence supports booster dosing as an important component of sustained protection. The evidence base is promising but still compact; ongoing phase 3 follow-up, special-population trials, and post-deployment effectiveness studies will be essential in defining durability, severe malaria protection, safety, and real-world impact.

## Figures and Tables

**Table 1:** Risk of bias in the independent randomized field-efficacy trials included in the primary meta-analysis.

Study	Randomization and Deviations	Outcome Data and Measurement	Reporting and Overall Judgment
Datoo et al. [23]	Low risk: randomized, double-blind, controlled trial with concealed allocation and prespecified groups.	Low risk: limited attrition; laboratory-confirmed clinical malaria and standardized follow-up.	Low risk: registered trial with prespecified efficacy analyses and concordant reporting.
Datoo et al. [22]	Low risk: multicentre, double-blind, randomized 2:1 allocation with masking of participants, families, investigators, laboratory teams, and study staff.	Low risk: objective clinical malaria case definitions and prespecified time-to-event analyses; high follow-up completeness.	Low risk: registered phase III trial with prespecified co-primary endpoints and transparent reporting.

**Table 2:** Characteristics and contributions of the eleven studies included in the systematic review.

Study	Design and Setting	Population/Exposure	Primary Contribution to This Review
**Field-efficacy randomized controlled trials**			
Datoo et al. [23]	Phase 2b double-blind RCT; Nanoro, Burkina Faso	Children aged 5–17 months; 5 micrograms of R21 with 25 or 50 micrograms of Matrix-M	Primary-series field efficacy, safety, and anti-NANP immunogenicity
Datoo et al. [22]	Multicentre double-blind phase III RCT; Burkina Faso, Mali, Kenya, and Tanzania	4878 children aged 5–36 months; seasonal and standard/perennial settings	Large-scale field efficacy, safety, immunogenicity, and transmission-setting effects
**Booster follow-up/durability**			
Datoo et al. [21]	Booster follow-up of the Nanoro phase 2b RCT	409 children returning for dose 4	Durability, 12-month post-booster efficacy, multiple episodes, and antibody correlates
**Early-phase safety, dose, and co-administration studies**			
Sang et al. [26]	Phase 1b age-de-escalation/dose-escalation study; Kilifi, Kenya	Adults, children, and infants; varied R21/Matrix-M doses	Longer-term safety and immunogenicity across age and dose groups
Hanboonkunupakarn et al. [25]	Phase 2 open-label, computer-randomized controlled trial; Thailand	120 healthy adults; R21/Matrix-M with or without antimalarial drugs	Safety and immunogenicity during antimalarial co-administration
Venkatraman, Tiono, et al. [27]	Two phase 1 first-in-human trials; UK and Burkina Faso	Malaria-naive and malaria-exposed adults; dose-escalation regimens	Early safety, dose selection, and immunogenicity
**Controlled human malaria infection (CHMI) studies**			
Venkatraman, Silman, et al. [24]	Open-label, partially blinded phase 1-2a CHMI study; UK	Malaria-naive adults; multiple schedules followed by mosquito-bite challenge	Controlled-challenge protection and schedule evaluation
Kapulu et al. [28]	Randomized phase 2b CHMI trial; Kenya	R21/Matrix-M-vaccinated participants challenged intradermally or intravenously	Route-specific controlled-challenge protection and mechanistic interpretation
**Mechanistic/secondary immunological studies**			
Bundi et al. [29]	Secondary immunological analysis of phase 1b samples; Kenya	Adults, children, and infants	Antibody magnitude, durability, complement fixation, avidity, and sporozoite-inhibition function
McDaniel et al. [11]	Mechanistic antibody repertoire analysis; UK trial samples	10 malaria-naive adult vaccinees	Polyclonal anti-NANP repertoire, cross-reactive epitopes, and functional monoclonal antibodies
**Qualitative/implementation study**			
Diawara et al. [30]	Qualitative implementation study nested within the phase III trial; Mali	Caregivers, community members, health workers, and trial staff	Community acceptability, trust, perceived efficacy, and interaction with SMC

**Table 3:** Field-efficacy data included in the meta-analysis of R21/Matrix-M against the first episode of clinical malaria.

Study (Design, Follow-Up)	Field Endpoint and Population	R21/MM *n*/*N*	Control *n/N*	HR (95% CI)
Datoo et al. [23] Phase 2b RCT, 12 months	First laboratory-confirmed clinical malaria episode; children aged 5–17 months in Nanoro, Burkina Faso; 5 micrograms of R21 + 50 micrograms of Matrix-M	39/146	106/147	0.23 (0.16–0.33)
Datoo et al. [22] Phase III RCT, 12 months	First clinical malaria episode; children aged 5–36 months at five sites in Burkina Faso, Mali, Kenya, and Tanzania	464/3102	627/1541	0.27 (0.24–0.31)

Note: HR, hazard ratio; CI, confidence interval. Vaccine efficacy was calculated as VE = 1 − HR. Approximate inverse-variance weights were 11.1% for Datoo et al. [23] and 88.9% for Datoo et al. [22]. The pooled HR was 0.265 (95% CI 0.235–0.299), corresponding to pooled vaccine efficacy of 73.5% (95% CI 70.1–76.5%). The Datoo et al. [21] booster extension was excluded from pooling because it represented continued follow-up of the same phase 2b cohort; CHMI studies were not pooled with natural-exposure field-efficacy trials.

**Table 4:** Field-efficacy estimates and their roles in the primary, sensitivity, and durability analyses.

Study/Estimate	Setting	Follow-Up	HR (95% CI)	VE	Analysis Role
Datoo et al. [23] high-adjuvant phase 2b	Seasonal, Burkina Faso	12 months after primary series	0.23 (0.16–0.33)	77% (67–84)	Primary + sensitivity
Datoo et al. [22] phase III overall	Multicentre African sites	12 months after primary series	0.27 (0.24–0.31)	73%	Primary trial-level
Datoo et al. [22], seasonal stratum	Seasonal phase III sites	12 months after primary series	0.25 (0.21–0.29)	75% (71–79)	Both analysis
Datoo et al. [22] standard/perennial stratum	Standard/perennial phase III sites	12 months after primary series	0.32 (0.26–0.39)	68% (61–74)	Both analysis
Datoo et al. [21] high-adjuvant booster	Linked Nanoro cohort	12 months post-booster	0.20 (0.15–0.28)	80% (72–85)	Descriptive durability

Note: HR, hazard ratio; CI, confidence interval; VE, vaccine efficacy. Vaccine efficacy was calculated as VE = (1 − HR) × 100%. The 2022 booster estimate is repeated follow-up of the phase 2b cohort and was not entered as an independent pooled estimate. The phase III seasonal and standard/perennial estimates were used only in sensitivity analyses and were not counted as separate independent trials in the primary model.

**Table 5:** Summary of primary and sensitivity meta-analysis results.

Analysis	Units	Model	Pooled HR (95% CI)	VE (95% CI)	I^2^
Primary trial level	2 independent trials	Random effects	0.265 (0.235–0.299)	73.5% (70.1–76.5)	0%
Primary trial level	2 independent trials	Fixed effect	0.265 (0.235–0.299)	73.5% (70.1–76.5)	0%
Transmission-strata sensitivity	3 non-overlapping estimates	Random effects	0.270 (0.223–0.326)	73.0% (67.4–77.7)	53.8%
Phase III-only sensitivity	2 non-overlapping strata	Random effects	0.281 (0.220–0.357)	71.9% (64.3–78.0)	71.3%

Note: The fixed-effect trial-level estimate is numerically identical to the random-effects estimate because tau^2^ = 0. For the transmission-strata sensitivity analysis, Q = 4.33 (*p* = 0.115; tau^2^ = 0.0151); for the phase III-only analysis, Q = 3.49 (*p* = 0.062; tau^2^ = 0.0217).

**Table 6:** Structured comparison of the R21/Matrix-M and RTS, S/AS01 evidence bases.

Evidence Domain	R21/Matrix-M Evidence	RTS, S/AS01 Evidence
Field efficacy	Two independent field-efficacy RCTs contribute to this meta-analysis: a phase 2b trial and a multicountry phase III trial.	The evidence base is larger and more mature, including multicountry phase III development and subsequent implementation evaluations.
Age groups	Pivotal field evidence is concentrated in young children, including those aged 5–36 months in the phase III trial; early-phase studies also include adults and other paediatric groups.	Clinical-development and implementation evidence spans infants and young children.
Follow-up/durability	The primary pooled endpoint is 12 months; booster follow-up extends to approximately 24 months in the phase 2b cohort.	Longer-standing clinical-development and post-introduction follow-up are available.
Severe malaria	Severe-malaria events are sparse; the phase III estimate was 67% but imprecise and not statistically significant.	A broader evidence base includes severe-malaria outcomes and implementation data, although estimates vary by schedule and setting.
Programmatic experience	Seasonal and age-based strategies have been evaluated; large-scale implementation and economic evidence are still accumulating.	Longer programmatic experience is available from pilot and routine implementation programmes.

Policy interpretation: the higher point efficacy observed for R21/Matrix-M should not be interpreted as proof of overall superiority. RTS, S/AS01 has greater evidence depth and longer programmatic follow-up, whereas R21/Matrix-M has a promising but less mature evidence base. Head-to-head or standardized comparative-effectiveness studies with matched age groups, transmission settings, booster schedules, and severe-malaria endpoints are needed for firmer policy comparisons [7].

## Data Availability

The quantitative data used in the meta-analysis are reported in Table 3 and are derived from the cited primary studies. The structured data-extraction form and analytic code used to reproduce the meta-analysis and forest plots are available from the corresponding authors upon reasonable request.

## Supplementary Materials

The following supporting information can be downloaded at: https://www.mdpi.com/article/10.3390/vaccines14090824/s1, Supplementary Table S1 provides the completed PRISMA 2020 checklist, and Supplementary Table S2 provides the database-specific search strategies and date limits used for this review. Supplementary Table S3 provides the detailed data extraction and summary of selected studies included in this systematic review.

## Abbreviations

The following abbreviations are used in this manuscript:

RCT	Randomized controlled trial
MeSH	Medical Subject Headings
PfSPZ	*Plasmodium falciparum* sporozoites
PRISMA	Preferred Reporting Items for Systematic reviews and Meta-Analyses
CSP	Circumsporozoite protein
SMC	Seasonal malaria chemoprevention
ACTs	Artemisinin-based combination therapies
SAE	Serious adverse event
