# Supplementary material for: Efficacy and Response of R21/Matrix-M in Malaria Vaccine: A Systematic Review and Meta-Analysis of Clinical Trials
Source: Vaccines (Basel). 2026 Sep 19;14(9):824. doi: 10.3390/vaccines14090824 (PMC13611381; doi:10.3390/vaccines14090824)
Supplement: Supplementary file 1 [file vaccines-14-00824-s001.zip › vaccines-4566090-supplementary.pdf]

Supplementary Materials

Supplementary Table S1. Completed PRISMA 2020 checklist for this systematic review and meta-analysis.

PRISMA topic	Item	How addressed in this review	Location
Title	1	Identify the report as a systematic review.	Title
Abstract	2	Report the principal rationale, information sources/time window, eligibility and synthesis methods, main results, limitations, conclusions, and review status.	Abstract
Introduction - rationale	3	Explain why the review is needed in the context of existing evidence.	Section 1
Introduction - objectives	4	State the review objective(s) and outcomes of interest explicitly.	End of Section 1
Eligibility criteria	5	Define inclusion/exclusion criteria and how studies were grouped for synthesis.	Section 2.3
Information sources	6	List databases and other sources consulted and state when they were last searched.	Section 2.2; Table S2
Search strategy	7	Provide database-specific search syntax, operators, and date limits.	Table S2
Selection process	8	Describe title/abstract and full-text screening, reviewer independence, and disagreement resolution.	Section 2.4
Data collection process	9	Describe structured extraction, number of reviewers, and consensus procedure.	Section 2.5
Data items - outcomes	10a	Define outcome domains and indicate which time point was selected for the primary meta-analysis.	Section 2.5
Data items - other variables	10b	List participant, intervention, design, setting, follow-up, registration, and funding variables collected.	Section 2.5
Risk of bias methods	11	Describe the RoB 2 tool, domains, and reviewer process for pooled randomized trials.	Section 2.7
Effect measures	12	Define hazard ratio and vaccine efficacy transformation used in quantitative synthesis.	Section 2.8
Synthesis eligibility	13a	Explain which studies were eligible for each synthesis and how overlapping reports were handled.	Sections 2.3 and 2.8
Data preparation	13b	Describe log-HR transformation and derivation of standard errors from confidence intervals.	Section 2.8
Display methods	13c	Describe use of structured tables and forest plots.	Section 2.6; Tables 1-5; Figures 2-5

Synthesis model	13d	Describe inverse-variance pooling, random/fixed models, heterogeneity statistics, and analysis software.	Sections 2.6 and 2.8
Heterogeneity exploration	13e	Describe the transmission-strata analysis used to explore setting-related variation.	Section 2.8
Sensitivity analyses	13f	Describe fixed-effect and phase III-only sensitivity analyses.	Section 2.8
Reporting-bias methods	14	State whether and why funnel-plot/regression methods were or were not used.	Section 2.8
Certainty methods	15	State the approach to confidence/certainty; formal GRADE was not performed.	Section 2.8
Study selection results	16a	Report numbers identified, deduplicated, screened, retrieved, excluded, and included using a flow diagram.	Section 2.4; Figure 1
Excluded reports	16b	Report reasons for full-text exclusion. Aggregate categories and counts are presented.	Section 2.4; Figure 1
Study characteristics	17	Present key design, population, exposure, setting, and contribution of each included study.	Table 2
Risk of bias results	18	Present RoB 2 judgments for each pooled randomized field-efficacy trial.	Sections 3.1 and 3.3; Table 1
Individual-study results	19	Present study-level data and effect estimate with precision where applicable.	Sections 3.2-3.4; Table 3; Figures 2-5
Synthesis context	20a	Summarize characteristics and risk of bias of studies contributing to each quantitative synthesis.	Sections 3.3-3.4
Synthesis results	20b	Report pooled estimates, confidence intervals, heterogeneity, and direction of effect.	Sections 3.4.1-3.4.2; Figures 2-4; Table 5
Heterogeneity results	20c	Present findings from transmission-setting analyses.	Section 3.4.2
Sensitivity results	20d	Present all sensitivity-analysis results.	Section 3.4.2; Figures 3-4; Table 5
Reporting biases	21	State that statistical reporting-bias assessment was not feasible with two independent trials.	Section 3.5
Certainty of evidence	22	State that formal grading was not performed and summarize factors informing confidence.	Section 3.5
Discussion - interpretation	23a	Interpret findings in relation to the wider malaria-vaccine evidence base.	Section 4
Discussion - evidence limits	23b	Discuss limitations of the included evidence.	Section 4.1

Discussion - review limits	23c	Discuss limitations of the review methods and synthesis process.	Section 4.1
Discussion - implications	23d	Discuss implications for practice, policy, implementation, and future research.	Sections 4 and 5
Registration	24a	State registration information or non-registration.	Section 2.1; Abstract
Protocol	24b	State where the protocol is available or that no separate protocol was prepared.	Section 2.1; Abstract
Protocol amendments	24c	Describe amendments or state that none apply.	Section 2.1
Support	25	Report funding source/grant and the role of the funder.	Funding statement
Competing interests	26	Declare competing interests.	Conflicts of Interest statement
Data/code/materials	27	State availability of extracted quantitative data, extraction form, and analytic code.	Data Availability Statement

Supplementary Table S2. Database-specific search strategies and date limits.

Source	Search strategy / approach	Limits / last searched
PubMed	("R21" OR "R21/Matrix-M" OR "R21 Matrix-M" OR "R21 malaria vaccine") AND (malaria OR "Plasmodium falciparum") AND (efficacy OR immunogenicity OR safety OR antibody OR "clinical trial")	Publication period: Jan 2021-Feb 2026; last searched Feb 2026
Scopus	TITLE-ABS-KEY(("R21" OR "R21/Matrix-M" OR "R21 Matrix-M") AND (malaria OR "Plasmodium falciparum") AND (efficacy OR immunogenicity OR safety OR antibody OR "clinical trial"))	Publication period: Jan 2021-Feb 2026; last searched Feb 2026
Embase	("R21":ti,ab,kw OR "R21/Matrix-M":ti,ab,kw OR "R21 Matrix-M":ti,ab,kw) AND (malaria:ti,ab,kw OR "Plasmodium falciparum":ti,ab,kw) AND (efficacy:ti,ab,kw OR immunogenicity:ti,ab,kw OR safety:ti,ab,kw OR antibody:ti,ab,kw OR "clinical trial":ti,ab,kw)	Publication period: Jan 2021-Feb 2026; last searched Feb 2026
Web of Science	TS=(("R21" OR "R21/Matrix-M" OR "R21 Matrix-M") AND (malaria OR "Plasmodium falciparum") AND (efficacy OR immunogenicity OR safety OR antibody OR "clinical trial"))	Publication period: Jan 2021-Feb 2026; last searched Feb 2026

Cochrane CENTRAL	("R21" OR "R21/Matrix-M" OR "R21 Matrix-M") AND (malaria OR "Plasmodium falciparum") AND (efficacy OR immunogenicity OR safety OR antibody OR "clinical trial")	Publication period: Jan 2021-Feb 2026; last searched Feb 2026
Other sources	WHO reports/publications and institutional publications/websites of the Jenner Institute and Serum Institute of India were consulted using the terms R21, R21/Matrix-M, Matrix-M, and malaria vaccine.	Consulted through Feb 2026; no additional eligible records included

Table S3. Data extraction and summary of selected studies

Authors, Years, and Region	Aims	Study Design, Sample Population	Vaccination Dosage Details	Results	Conclusion and Limitations
Datoo et al. [22]<br><br>Burkina Faso	To assess the safety, immunogenicity, and efficacy of the malaria vaccine candidate R21 adjuvanted with Matrix-M (R21/MM) in young children	A double-blind, randomized, controlled phase 2b clinical trial among 498 children aged 5–17 months	• Group 1 received 5µg R21 plus 25µg Matrix-M adjuvant.<br>• Group 2 received 5µg R21 plus 50µg Matrix-M adjuvant.<br>• Group 3 received control rabies vaccine.	• Clinical malaria occurred in 29% (43/146) in group 1, 26% (38/146) in group 2, and 71% (105/147) in control group.<br>• Vaccine efficacy remained high at 77% (67–84) for group 2 (5µg R21 + 50µg MM).	• R21/MM vaccine shows promising high-level efficacy (≥75%) against clinical malaria in African children in a high transmission setting, meeting the WHO target for malaria vaccine development.<br>• The trial was conducted in a single site with highly seasonal malaria transmission, which may limit generalisability.
Datoo et al [21]	To evaluate the safety, immunogenicity,	Phase 1/2b double-blind randomized controlled trial of	• Primary vaccination: three doses of 5µg R21 with either 25µg (Group 1) or 50µg	• Low-dose adjuvant group: 71% efficacy (95% CI 60–78; p<0.0001).	• R21/Matrix-M vaccine meets the WHO goal of at least 75% vaccine

Authors, Years, and Region	Aims	Study Design, Sample Population	Vaccination Dosage Details	Results	Conclusion and Limitations
Burkina Faso	and long-term efficacy (over 2 years) of the R21/Matrix-M malaria vaccine in young children living in a high seasonal malaria transmission area	450 children aged 5–17 months.	(Group 2) Matrix-M adjuvant.<br>• Booster vaccination with the same dose administered 12 months later.<br>• Control group received rabies vaccine (Rabivax-S) on the same schedule	• High-dose adjuvant group: 80% efficacy (95% CI 72–85; p<0.0001).<br>• Significant difference in efficacy between low and high-dose groups (HR 0.68, 95% CI 0.48–0.98; p=0.038)	efficacy against clinical malaria sustained over 2 years in African children in a highly seasonal transmission setting.<br>• However, limited power to fully identify and validate correlates of protection was a limitation.
Sang et al. [26]<br><br>Kenya	To evaluate the safety and immunogenicity of different doses of the R21 malaria vaccine with the Matrix-M adjuvant.	• Phase 1b, open-label, age de-escalation, dose-escalation trial.<br>• Total of 91 participants enrolled out of 188 screened, after exclusion.	Three regimens tested:<br>• 10µg R21 + 50µg Matrix-M,<br>• 5µg R21 + 25µg Matrix-M,<br>• 5µg R21 + 50µg Matrix-M.<br>• Vaccine was administered as a three-dose primary vaccination series doses spaced 4 weeks apart.<br>• Boosters given 1-2 years after the primary series.	• The vaccine was safe and well tolerated with mostly mild and self-limiting adverse events such as local injection site reactions and mild systemic symptoms.<br>• Peak immune response was observed in infants 28 days after the third dose of the primary series.	R21/Matrix-M vaccine is safe and immunogenic at varied doses as it induces robust antibody responses, especially in infants - the target group.
Datoo et al. [23]<br><br>Five sites of African countries	To evaluate the efficacy, immunogenicity, and safety of the	• Phase 2b/3 randomized, controlled, observer-blinded trial conducted	• Children received three primary doses of the R21/Matrix-M vaccine, administered at intervals of 3–16 weeks	• Overall vaccine efficacy against clinical malaria was 73% (95% CI 70–76%) over 12 months. Efficacy was	The R21/Matrix-M vaccine demonstrates high efficacy (~73%) in preventing clinical

Authors, Years, and Region	Aims	Study Design, Sample Population	Vaccination Dosage Details	Results	Conclusion and Limitations
	R21/Matrix-M malaria vaccine	across multiple sites in Africa.<br>• A total of 5477 children aged 5 to 36 months were enrolled and screened. Among these, 5139 children were randomly assigned equally to either the R21/Matrix-M vaccine group (n=3434) or the control group (n=1705).<br>•	(median approximately 14 weeks).<br>• A booster dose was also given. The control group received a comparator vaccine.	slightly higher in seasonal sites (~75%) compared to standard sites (~68%).<br>• The vaccine also significantly reduced asymptomatic parasitaemia at 12- and 18-months post-vaccination ($p<0.0001$ and $p=0.0014$, respectively).<br>• Quantitative IgG responses against NANP repeats increased significantly post-vaccination, correlating with protective efficacy.	malaria in children aged 5–36 months, with a favorable safety profile. However, longer-term durability beyond 2 years remains to be established.
Hanboonkunupakarn et al. [25]<br><br>Thailand	To assess the tolerability, safety, and immunogenicity of the malaria vaccine R21/Matrix	Phase 2, open-label, computer-randomised, controlled safety and immunogenicity trial.	• Group 1 (n=50): Received R21/Matrix-M with 3 days of dihydroartemisinin-piperaquine and a single low dose of primaquine (SLDPQ) concurrently.<br>• Group 2 (n=50): Received R21/Matrix-M vaccine alone without antimalarials.<br>• Group 3 (n=20): Received antimalarial	• The R21/Matrix-M vaccine was safe and well tolerated, both alone and with antimalarials.<br>• No severe adverse events attributable to the vaccine or drug combination were observed.<br>• Immunogenicity, assessed through antibody responses, showed comparable	R21/Matrix-M malaria vaccine was safe, well-tolerated, and immunogenic.

Authors, Years, and Region	Aims	Study Design, Sample Population	Vaccination Dosage Details	Results	Conclusion and Limitations
			drugs only without vaccination	and high responses in both groups receiving the vaccine, with or without antimalarial drugs.	
Bundi et al. [29]<br><br>Kenya	To evaluate how age, vaccination dose, and prior malaria exposure impact the magnitude, quality, and function of immune responses induced by the R21/Matrix-M malaria vaccine among healthy volunteers	• Phase 1b, open-label, age de-escalation, and dose-escalation trial.<br>• Total of 91 participants: adults (mean age ~28 years, n=18), children (~3 years, n=17), and infants (~7 months, n=51).	• Different doses of R21 antigen (5 µg) and Matrix-M adjuvant (25 µg or 50 µg) given, particularly in infants, to assess dose effect.<br>• A subgroup received a high adjuvant dose (50µg Matrix-M) versus low adjuvant dose (25µg) to evaluate immune response quality and magnitude.	• R21/Matrix-M vaccine induced robust IgG antibodies to full-length R21, NANP, C-terminus, and Hepatitis B surface antigen components significantly increased from baseline to Day 84 ($p < 0.0001$).<br>• No significant difference in antibody levels between different vaccine/adjuvant doses in infants was observed, likely due to small sample size (~15 per group).	R21/Matrix-M induces strong humoral immune responses in malaria-exposed populations with variations by age. However, limited mechanistic understanding of age-related differences in vaccine response remains.
Venkatraman, Silman, et al. [24]<br>United Kingdom	To evaluate the safety, immunogenicity, and efficacy of various dosing regimens of the R21 malaria vaccine candidate adjuvanted with	• This was an open-label, partially blinded, phase 1–2A controlled human malaria infection (CHMI) study.<br>• A total of 66 healthy adult volunteers (both	• Standard three-dose regimen: 10µg R21 + 50µg Matrix-M per dose, administered at monthly intervals (groups 1 and 6).<br>• Reduced (fractional) third dose regimens include either 50µg, 50µg, and 10µg (group 2), or 10µg, 10µg, and	• Highest protection observed in the standard three-dose regimen of R21/MM: 9 of 11 volunteers were protected against CHMI (approx. 80% protection), maintained in 3 of 5 volunteers upon rechallenge 7.5 months later.	• The R21 malaria vaccine adjuvanted with Matrix-M is safe, well tolerated, and demonstrates high-level efficacy against controlled human malaria infection in malaria-naive

Authors, Years, and Region	Aims	Study Design, Sample Population	Vaccination Dosage Details	Results	Conclusion and Limitations
	Matrix-M (R21/MM) against controlled human malaria infection (CHMI) in malaria-naive adults	men and non-pregnant women), aged 18–45 years, malaria-naive, were enrolled, with 59 undergoing CHMI.	2µg (group 5) of R21 with the standard Matrix-M amount.	• The reduced third dose groups showed comparable efficacy (group 2: 63%, group 5: 77%), but no statistically significant improvement over standard regimens.	adults following a three-dose regimen.<br>• The study had a small sample size and limited statistical power, typical of CHMI trials that are designed as proof-of-concept studies.
Venkatraman, Tiono, et al. [27]<br>United Kingdom and Burkina Faso	To assess the safety, tolerability, and immunogenicity of the malaria vaccine candidate R21, administered with or without the Matrix-M adjuvant, in adults	• Phase 1a: Open-label safety and immunogenicity trial in the UK on malaria-naïve 31 adults.<br>• Phase 1b: Randomised controlled trial in Burkina Faso on 13 adults from a malaria-endemic region.	• Phase 1a groups:<br>• 50µg R21 only (no adjuvant)<br>• 50µg R21 with 50µg Matrix-M<br>• 10µg R21 with 50µg Matrix-M<br>• 2µg R21 with 50µg Matrix-M (added later based on prior immunogenicity findings)<br>• Phase 1b groups:<br>• 10µg R21 with 50µg Matrix-M<br>• Saline placebo	• R21/Matrix-M induced strong IgG responses to the NANP repeat region of CSP, considered a correlate of protection.<br>• Antibody levels induced by R21/Matrix-M were similar to those previously associated with protection by RTS,S/AS01 even at 5- to 25-fold lower antigen doses.<br>• T-cell responses (IFN-γ ELISpot) after vaccination were weak but similar between R21 and RTS,S recipients	R21 delivered with Matrix-M is safe and well tolerated in both malaria-naive adults and semi-immune adults.
Diawara et al. [30]	To investigate the acceptability of the	A qualitative study utilizing in-depth interviews (IDIs),	• Children received three monthly doses of the R21/Matrix-M vaccine	• The community perceived high efficacy, with observations that	The R21/Matrix-M vaccine was found to be acceptable among

Authors, Years, and Region	Aims	Study Design, Sample Population	Vaccination Dosage Details	Results	Conclusion and Limitations
Mali	R21/Matrix-M malaria vaccine among caregivers and community members	focus group discussions (FGDs), and exit interviews. The study involved 72 IDIs, 20 FGDs, and 45 exit interviews; total respondents numbered 299.	or control (rabies vaccine) in May–July 2021.<br>• An additional booster dose was administered in June 2022.<br>• The trial used a 2:1 randomization ratio favoring the R21/Matrix-M vaccine.	children in the trial were healthier and recovered quickly from illnesses.<br>• The perceived effectiveness was influenced by the high efficacy reported in the trial and the community's observations.<br>• Attitudes towards the vaccine were positive, driven mainly by the high malaria burden in the area.	caregivers and community members, largely due to the high malaria burden and trust in the trial team. However, the potential influence of circulating rumors and misinformation, especially related to COVID-19, which could impact vaccine acceptability.
(Kapulu et al. [28]<br><br>Kenya	To test whether the protective efficacy of R21/Matrix-M varies according to the route of Plasmodium falciparum sporozoite inoculation, specifically intradermal (ID) versus direct venous injection (DVI), using a controlled human malaria infection (CHMI) model.	• Phase 2b, open, randomized controlled human malaria challenge trial conducted in healthy volunteers in Kenya.<br>• A total of 135 volunteers were screened; 80 eligible volunteers were randomized and vaccinated.<br>• Volunteers were randomized to R21/Matrix-M (n=38), ME-TRAP-based vaccines	• R21/Matrix-M recipients received three intramuscular vaccinations at 4-week intervals.<br>• Each R21 dose contained 10 µg R21 mixed with 50 µg Matrix-M adjuvant.<br>• CHMI was performed 4 weeks after the final vaccination.<br>• ID PfSPZ challenge dose was 22,500 total sporozoites, delivered as 11,250 PfSPZ in each arm; DVI PfSPZ challenge dose was 3,200 sporozoites.	• No R21 ID volunteer met the prespecified primary endpoint, whereas 5/5 R21 DVI volunteers met the primary endpoint ($P<0.0005$ by log-rank across groups).<br>• Seven of 8 control ID volunteers (88%) and 11/12 ME-TRAP ID volunteers (92%) met the primary endpoint.<br>• Secondary efficacy endpoints showed the same pattern: R21 ID volunteers did not meet secondary parasitemia endpoints, while other groups met endpoints between days 7 and 21.	• R21/Matrix-M was highly protective against ID PfSPZ challenge but showed no evidence of protection against DVI PfSPZ challenge.<br>• The findings suggest that anti-CSP antibody correlates of protection should be assessed separately for ID and venous sporozoite challenge routes.<br>• Limitations include the small R21 DVI sample size, reliance on historical DVI controls,

Authors, Years, and Region	Aims	Study Design, Sample Population	Vaccination Dosage Details	Results	Conclusion and Limitations
		(n=24), or control (n=18).<br>• Thirty-seven volunteers completed CHMI with PCR monitoring of parasitemia; key challenged groups included R21 ID (n=12), R21 DVI (n=5), ME-TRAP ID (n=12), and control ID (n=8). Historical DVI controls (n=34) were also compared.		• Postvaccination local and systemic adverse events were uncommon; no serious or severe adverse events were reported.<br>• Sensitivity analysis still suggested substantial ID protection (9/12; 75%) when one-off low-density PCR-positive signals were counted as exploratory endpoints.	and the fact that needle-based ID/DVI challenge may not fully reproduce infectious mosquito-bite exposure.
